# Single-Point and Surface Quality Assessment Algorithm in Continuous Production with the Use of 3D Laser Doppler Scanning Vibrometry System

**DOI:** 10.3390/s23031263

**Published:** 2023-01-22

**Authors:** Lukasz Scislo

**Affiliations:** Faculty of Electrical and Computer Engineering, Cracow University of Technology, Warszawska 24, 31-155 Cracow, Poland; lukasz.scislo@pk.edu.pl

**Keywords:** QA, QC, EMA, LDV, laser doppler vibrometer, modal analysis, quality control

## Abstract

In the current economic situation of many companies, the need to reduce production time is a critical element. However, this cannot usually be carried out with a decrease in the quality of the final product. This article presents a possible solution for reducing the time needed for quality management. With the use of modern solutions such as optical measurement systems, quality control can be performed without additional stoppage time. In the case of single-point measurement with the Laser Doppler Vibrometer, the measurement can be performed quickly in a matter of milliseconds for each product. This article presents an example of such quality assurance measurements, with the use of fully non-contact methods, together with a proposed evaluation criterion for quality assessment. The proposed quality assurance algorithm allows the comparison of each of the products’ modal responses with the ideal template and stores this information in the cloud, e.g., in the company’s supervisory system. This makes the presented 3D Laser Vibrometry System an advanced instrumentation and data acquisition system which is the perfect application in the case of a factory quality management system based on the Industry 4.0 concept.

## 1. Introduction

New hardware and software solutions for quality management (QM) in manufacturing are being introduced as a result of advances in novel instrumentation and data acquisition in conjunction with Industry 4.0 strategies for data management and analysis. The concept of QM is important not only for product development and prototyping [1] but also for product manufacturing [2], software solutions [3], and whole environments [4].

Using quality management and its methodology, any company can ensure that their manufactured products are of high quality and consistent. There are usually two distinct components to quality management: quality assurance (QA) and quality control (QC). Despite the similarities, there are some distinct differences between the two. QA is more concerned with how a process is accomplished or how a product is produced, while QC is more concerned with how the product is inspected. For both QA and QC, there are many options and strategies. Implementation depends on the type of production, system installation possibilities, time, space, and personnel limitations, etc. In general, every QA and QC strategy aims to achieve:

- The absence of manufacturing defects, limiting customers’ future complaints;
- No unexpected downtime increasing the production costs;
- No accidents caused by faulty equipment.

Software development for quality management purposes is also a key issue in the case of Industry 4.0 concept implementation in production companies. With the use of Internet of Things (IoT) sensors or sensor grids, it is possible to use cloud-based solutions for data analysis [5], artificial intelligence (AI) and machine learning (ML) applications [6,7], big data and data mining [8,9], and emerging blockchain technologies [10,11]. Additionally, the use of IoT sensors allows a continuous approach to predictive maintenance and production line fault detection [12]. Finally, due to the extensive use of vision systems, novel methods of visualization such as virtual reality can be used in connection with real-life data from IoT sensors [13].

In the case of manufacturing process QA and QC, depending on the measuring technique used the methods can be divided into destructive and non-destructive testing [14,15,16]. In the case of the continuous production process, non-destructive methods are especially interesting. One of the simplest QM systems based on non-contact solutions is using visual inspections with, e.g., high-speed cameras. This approach is often also connected with cloud solutions to analyze and compare data in production management systems [5]. Other optical-based solutions are based on using different optical scanners, digital image correlation tools, spectroscopy, tomography, and microscopy. Additionally, not only are image or video-based solutions used for the QA and QC applications but often the quality of the vision system is evaluated with sophisticated algorithms to improve the QA results as well as their perception by the human eye [17,18,19]. Another method of non-destructive testing is to use a microphone or microphone grids to check the quality of the product as well as to detect machine faults [20,21]. It is evident that the choice of solutions is wide, and the use of specific tools is determined by the process, product, time, and costs. One of the most versatile solutions is the possibility of using a 3D Laser Doppler Vibrometer (3D LDV) as an optical-based sensor. The 3D LDV can be especially useful for measurements of small, lightweight, or very fragile elements where other contact methods are not possible or will take significant time and personal involvement for testing. This method is appliable regardless of the material, thus can be appliable for testing elements obtained by additive manufacturing and especially composite materials [22,23].

The primary use of the 3D LDV in the industry is for materials, samples, components and whole systems testing. The system allows obtaining the data to confirm the finite element method (FEM) simulations or provides specific data on the material itself. In civil engineering and geoengineering, the method is used as an experimental way to establish a failure criterion in order to predict future rock failure [24]. In materials, science engineering 3D LDV is used for non-destructive material characterization, e.g., while using the impact excitation technique to calculate the elastic properties of a given sample using frequency response data [25]. However, in the case of material testing applications, most applications use modal analysis (MA) [22] and operational modal analysis (OMA) [26]. Another wider group of applications is for structural health monitoring. In this case, the 3D LDV is used as a means to check the dynamic conditions of civil engineering structures. Some applications include structural monitoring of large industrial facilities [27], monitoring of cooling and ventilation systems [28], and monitoring of renewable energy installations, e.g., wind turbines [29]. The third group of typical applications is quality testing in different industries. Most often, the system is utilized as a way to detect internal material errors, especially in composite or 3D-printed materials [30,31]. The method was also proven effective for prototype quality testing with a data connection to simulations of created virtual models [32]. The 3D LDV is also used for the inspection of especially problematic elements during production, e.g., welds and other connections [33]. However, in those cases, the application is usually limited to single samples, not serial production. The primary use examples for mass production quality check inspections can be found in the automotive industry [34]. In this case, the system is used for measurements of full surfaces or parts as stationary operations, which require some time to perform the measurement.

During the literature review and state-of-the-art cross check, it was found that there is no information about the possibility of using a 3D LDV system in mass production without stopping the process to perform the measurement. Thus, this paper focuses on the first two goals of the QA and QC strategy, which is finding a way to limit manufacturing defects and at the same time decrease possible downtime (for QA and eventual machine faults). These goals can be achieved with modern, advanced, and automated sensor technologies. Together with appropriate data collection and analysis (e.g., cloud solutions, big data, etc.), the system enables the creation of a reliable quality management system within Industry 4.0, whose main goal is to limit downtime in quality assessment. In particular, the non-destructive, non-contact method is a good solution for monitoring processes and products (according to ISO9000). As mentioned, one of the most advanced quality control and monitoring systems is the 3D Laser Doppler Vibrometer. This system, often used in the automotive industry [35], makes it possible to measure the frequency response of one or more points (surfaces) of a product or machine without downtime. Although the 3D LDV is used for measurement, existing implementations generally continue to use a contact method to energize the probed object (exciter). This is often achieved with an electrodynamic shaker or automatic modal hammer. Although this method is suitable for experimental modal analysis (EMA), it requires time and the influence of a quality control engineer. This paper describes a completely non-contact approach using sound pressure change as the excitation source (loudspeaker) and the 3D LDV (PSV-500, Polytec) as the measurement system. Additionally, the architecture of the multilayer usage of data is presented together with a QA comparison algorithm that can be used directly for cloud analysis solutions or in connection with other possible, presented solutions. As mentioned before, no direct applications of the system where no stopping of the production process is required were found, and no information about the possible architecture of such a system was mentioned in published studies. This is especially crucial taking into account the Industry 4.0 concept where users need to obtain as much data as possible from the process to optimize production and resources or to find new opportunities. Both elements—the procedure for QA and QC measurements without process delay and the architecture of the Industry 4.0 data acquisition and analysis system—are the key novelty components of this paper. This paper is an extension of the study presented at the 2021 11th IEEE International Conference on Intelligent Data Acquisition and Advanced Computing Systems: Technology and Applications (IDAACS), where a modal analysis of a metallic surface was presented [36].

## 2. Materials and Methods

### 2.1. Modal Analysis Techniques

**Figure 1:**
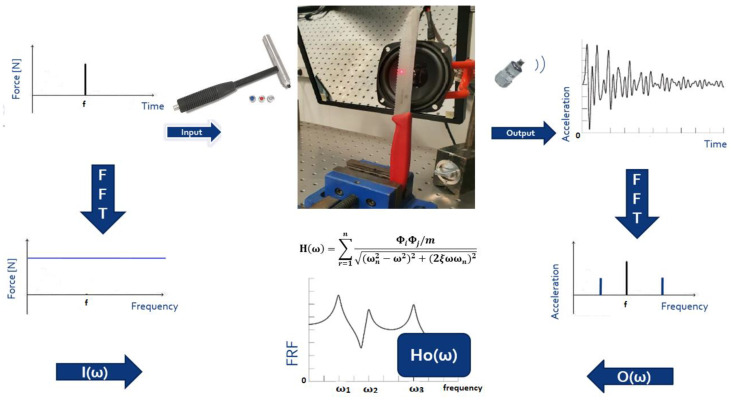
Modal analysis process schema.

The modal analysis involves determining the inherent dynamic characteristics of the system in terms of natural frequencies, damping factors, and mode shapes and using them to create a mathematical model of the dynamic behaviour of the system. Figure 1 presents the typical algorithm for obtaining modal data. A typical laboratory environmental measurement system should consist of three parts. As an example, consider a simple case with one input and one output. The first element of the measurement system is the one responsible for inputting the energy to the tested object or system. The second element aims to perform the measurement (sensor) and eventually to temporarily store the data (e.g., shocklog sensors). The last element allows signal processing and data presentation in the form of the frequency response function (FRF).

**Figure 2:**
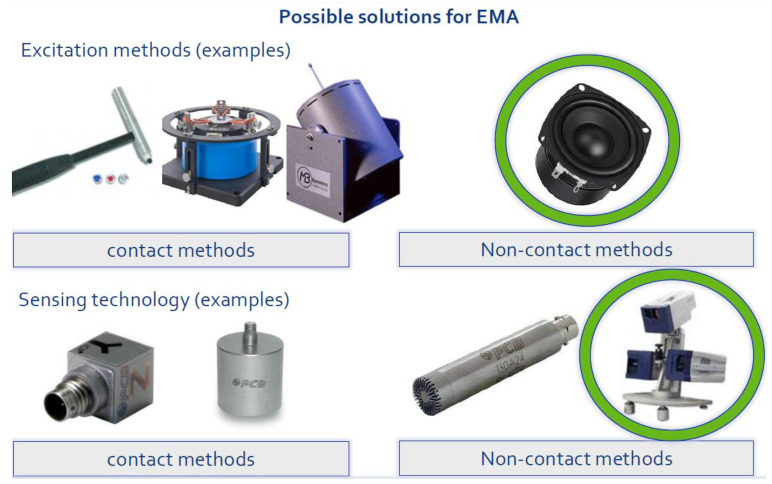
EMA possible solutions for sensing techniques and excitation sources.

Figure 2 shows typical equipment used as both an excitation source and a sensing element. In general, either contact and non-contact techniques or a mixture of both can be used. One of the most important steps in the measurement process is to select an excitation function (e.g., random noise) and an excitation system (e.g., a shaker) that is best suited to the application. When selecting the excitation, consider both the type of function desired and the type of excitation system available, as the two are interrelated. There are four main features to consider when selecting vibration meters and analyzers: accuracy, sampling frequency, and environmental conditions. The choice of equipment is particularly important in the case of QM. Moreover, the modern concepts of Industry 4.0 require that data are usually transmitted and analyzed in distributed systems. Therefore, the choice of devices must allow easy data extraction and communication with, e.g., cloud-based solutions.

In the following chapters, the concept of the contactless approach is presented. The loudspeaker as an element supplying the energy to the system (sound pressure) is used, and the PSV-500 (Polytec), a 3D Laser Doppler Vibrometer, is implemented as a sensing device.

### 2.2. The Principle of Operation of the 3D Laser Doppler Vibrometer

In addition to dynamic measurements of microstructures and large structures and buildings [37,38], the 3D Laser Vibrometry System also measures environmental conditions (temperature, electromagnetic field, radiation, etc.) and uses state-of-the-art technology for data analysis and postprocessing. The high cost of this technology means it is used mainly in the automotive industry [39], the aerospace industry [38], and for testing light and ultra-light structures [22,23].

An LDV is generally a type of velocity measurement system that utilizes the Doppler effect for its operation. When there is relative motion between a stationary observer and an oscillating source, the Doppler effect is the change in frequency of a wave motion.

It is easy to apply the above definition to the situation when a laser source is used to make the measurement (e.g., the laser used in an LDV) and the sample (object) moves at velocity v. In this case, the vibrometer is a source of frequency f. During measurement, the sample receives a frequency $f^{\prime }$ (1).

(1) $$f^{\prime }=\frac{\left(c-\vec{v}\;\cdot \;\vec{e_{t}}\right)}{c}f$$

Here, $\vec{e_{t}}$ is the unit vector of the transmitter (source), and c is the speed of waves in the given medium (in air, the speed of light). The velocity of the source is given relative to the chosen medium. Moreover, its value is positive when the source moves away from the receiver.

The examined target reflects the light beam and is the initial source of the frequency $f^{\prime }$. Lastly, the vibrometer sensor is the receiver of the frequency $f^{\prime \prime }$ (Equations (2) and (3)).

(2) $$f^{\prime \prime }=\frac{c}{\left(c-\vec{v}\;\cdot \;\vec{e_{r}}\right)}f^{\prime }$$

(3) $$f^{\prime \prime }=\left(\frac{c}{\left(c-\vec{v}\;\cdot \;\vec{e_{r}}\right)}\right)\left(\frac{c-\vec{v}\;\cdot \;\vec{e_{t}}}{c}\right)f\approx \left(1+\frac{\vec{v}\;\cdot \left(\vec{e_{t}}-\vec{e_{r}}\right)}{c}\right)f$$

Here, $\vec{e_{r}}$ is the receiver unit vector. It is described as the velocity of the receiver relative to the chosen medium. Consequently, it is positive when the receiver moves towards the light source (the sensor in the stationary measuring head of the LDV).

While the *c*>>v, the adjustment in frequency depends generally on the relative velocity of the original source and detector itself.

Moreover, if v is set parallel to the beam of the laser, the fractional change in the detected frequency becomes (4).

(4) $$\Delta f=\left(f^{\prime \prime }-f\right)=\frac{2v}{\lambda }$$

The presented Equations (1)–(4) are the usual way to explain, in an easy way, the working principle of the Doppler effect used for the laser-based sensors (LDV measurement equipment) [40,41].

Although the LDV is a complex device, in a direct 2D LDV setup it only measures the velocity component in the direct direction of the laser beam. When the velocity vector of the object and the normal of the wavefront form an angle θ (called the propagation or scattering angle), the frequency shift changes to (5).

(5) $$\Delta f=\frac{2\vec{v}\;\cdot \;\vec{e}}{\lambda }=\frac{2\;v_{x}}{\lambda }cos\theta$$

Using diffuse light from its target, the LDV measures velocity. As a result, the diffuse reflection characteristics and rotational angular displacement of the object will undoubtedly affect the final outcomes. When the observation direction changes angularly between the surface normal and the observation direction of the object (a Lambertian light object), diffuse reflection intensity follows the cosine law of the radiation source. A reduction in light intensity received by the dual LDVs is observed whenever an object experiences an angular displacement θ. Weakened light intensity is detected by the detectors of the LDVs. Signal-to-noise ratios also deteriorate as a consequence. The rotation of the target and the non-uniform reflectivity of the target surface lead to non-uniformity of the received light, which leads to a decrease in the signal-to-noise ratio [22]. Commercially available LDVs (such as Polytec’s PSV-500 used for this research) have a maximum relative measurement error of 2% (measurement range: ±10°) of angular displacement, which means that its absolute measurement error is only 0.2°. Moreover, the relationships observed for typical scattering angle values can be seen in (6).

(6) θ=2°⇒0.06% errorθ=8°⇒1% errorθ=45°⇒29.3% error

**Figure 3:**
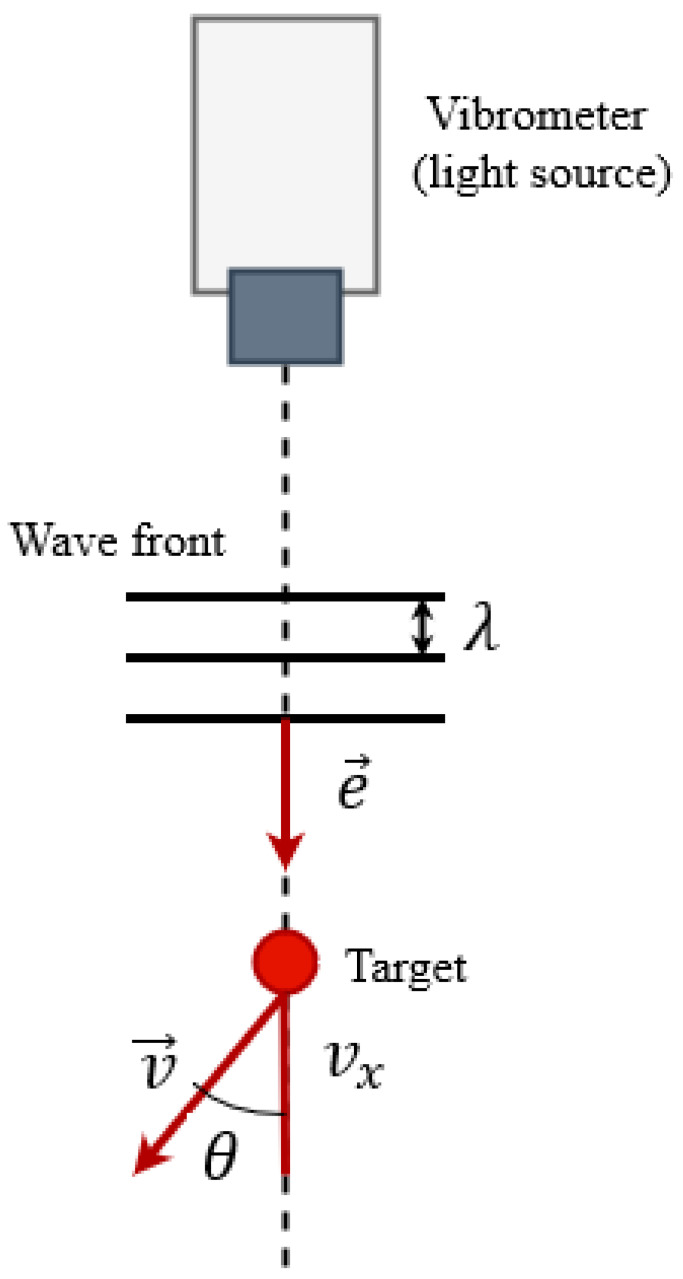
Vibrometer directionality and working principle scheme.

Finally, Figure 3 shows in detail the schema for the relations described by the equations above.

The system controller supplied with the 3D LDV allows all three measuring heads to perform and save the measurement and to use specialized software to perform three-dimensional velocity mapping. Finally, the results can be seen as a 3D virtual visualization of the mode shapes of the analyzed object. The system can be used both as a single-point measuring system and a multipoint one. If a quick scan is required, e.g., for a quality assurance test, the single-point scan can be performed in under 100 ms, with most systems’ limit going down to 30 ms. This allows incorporating the system in a chosen place on the production line and capturing the measurement of each product without process interruption. However, in many cases, to assure an even better quality control, often some products are chosen for detailed quality analysis. In this case, the chosen n-th object can be scanned using multipoint settings (surface scan or 3D 360° scan with the use of mirrors). Both approaches allow the detection of some specific changes in the production process that effect the change in quality of the product. An example of such a process will be shown in the next chapters.

### 2.3. Measurement Test Setup

The PSV-500 (manufactured by Polytec) is an LDV system that uses three separate Laser Doppler Vibrometers (three heads), each equipped with a set of two mirrors (vertical and horizontal), allowing the measuring laser to be moved over the multiple points assigned on the surface of the analyzed object. The laser used in the vibration measurement system is a HeNe laser (λ = 633 nm), and its working distance ranges from 125 mm (fixed laboratory mount) to about 100 m (field measurement stand). This allows high flexibility in measurements. With the measurement of a surface possible from just a few mm^2^ to a hundred m^2^, with a resolution of 0.01(μm/s)/√Hz (10 nm at 1 Hz) and frequency range of 0–100 kHz, the system is able to measure any kind of object. The measurement range from 0.001 to 10 m/2 also allows different excitation sources and, especially in the case of civil engineering usage, the use of only the ground motion as an excitation source. Under laboratory conditions where the environment is controlled and stable and there are no additional optical disturbances, vibrometers perform measurements with an error of <10^−5^. Moreover, the laser stabilization improves the overall signal-to-noise ratio which allows good-quality measurements in the industrial environment.

**Figure 4:**
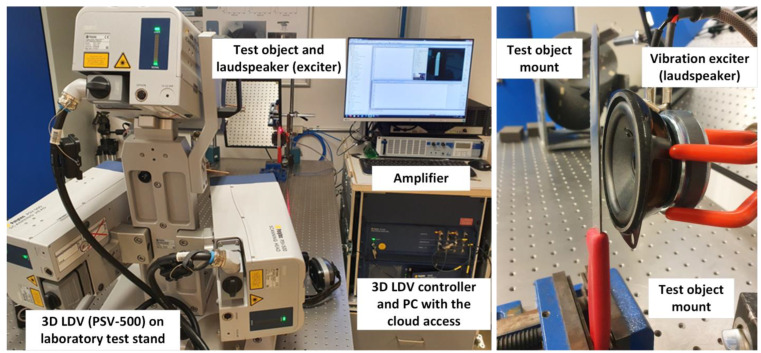
Illustration of experimental arrangement for a measurement.

Figure 4 shows the three heads of the 3D LDV in a laboratory test frame, a controller, and a computer with specialized data analysis software and cloud connection. The three heads can also be installed on a separate camera stand, in which case the system is completely mobile and ready to measure particularly large structures or machines.

The measurement test setup is presented in Figure 4. In this case, a commercial steel blade was the chosen object, which was clamped and fixed to the laboratory test table. The three heads of the PSV-500-3D system were used as sensing equipment, and the loudspeaker was the exciter used to deliver the energy to the object (with a specific function). The loudspeaker also requires a wave generator, which is integrated into the controller of the 3D LDV system. This allows, depending on the tested object and users’ needs, the generation of all typical waveforms, both standardized and random ones. Typically, the sweep or chirp signals are used; however, for testing with the use of the sound source (loudspeaker), this is not always practical. In this case, the pseudo-random signal is a much better choice. It can cover the entire frequency spectrum such as sweep or chirp signals but is less problematic for the eventual people around the setup due to the emitted sound. This choice is optimal for both laboratory testing and use in a production line environment.

**Figure 5:**
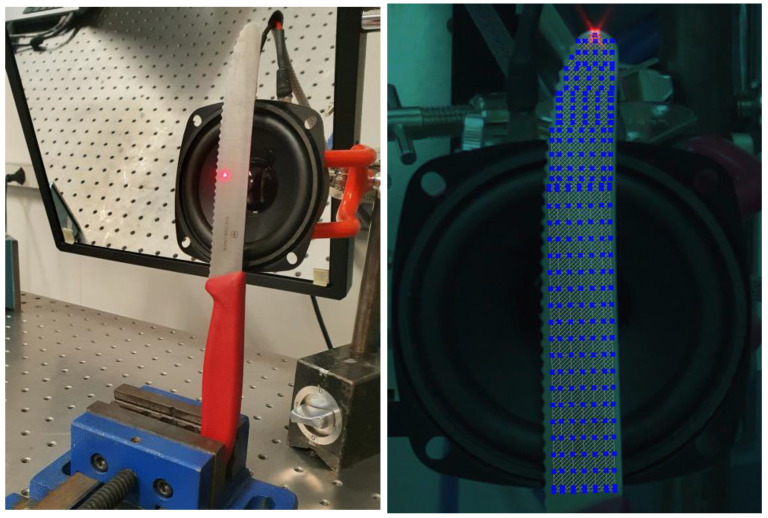
Test object with the exciter (loudspeaker) in a single-point measurement (**left**) and surface measurement—multiple points (**right**).

Regarding measurement performance, a single-point scan or multipoint scan is possible. If a single-point scan is chosen, the measurement can be performed in just 30 ms; however, it may be profitable to scan this point a few times to acquire the averaging parameter equal to the number of scans and improve the final results. In the case of a surface scan, a grid of assigned points is scanned. If for any reason the system excludes any of the points (low quality, lack of visibility, diffraction due to the dust in the air, etc.), the points are remeasured at the end of the scan. With the use of a loudspeaker, the full measurement can be performed in a matter of a few minutes. Figure 5 presents the laser focus on one point for single-point measurement (Figure 5, left) and assigned points for surface measurement in the case of a multipoint scan (Figure 5, right).

**Figure 6:**
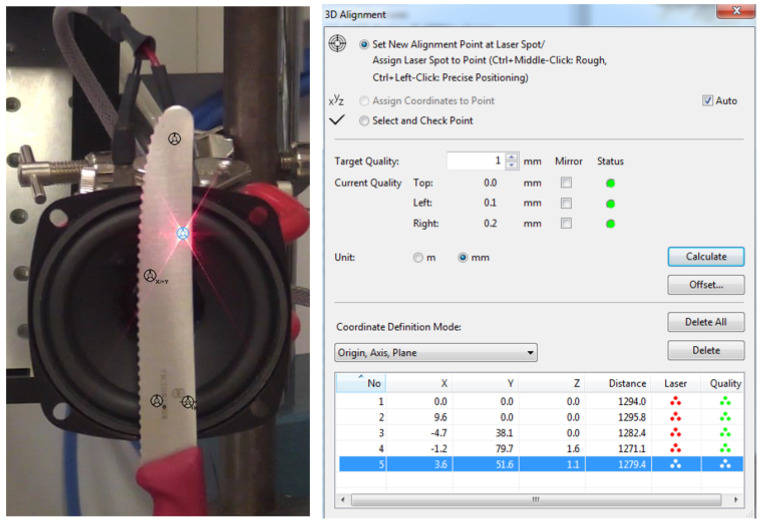
Test object coordinate system and 3 sensors’ (heads’) 3D alignment.

The system requires some preparatory steps to assure the quality of measurement without errors. This includes 2D alignment where each head distance is aligned to multiple points over the surface, 3D alignment where a coordinate system is created (Figure 6, left), assignment of scan points, and finally the geometry scan and beam focusing. For coordinate system creation (Figure 5) in the presented case, the X and Y directions are the lateral directions of the blade, and the vertical component Z is perpendicular to the surface (direction of the excitation). During this step, the system also checks the target quality and informs about visibility and eventual problems (Figure 6, right). For laboratory tests and industrial applications, the preferable target quality should be below 0.5 mm. In the case of the field test and especially large structures such as bridges or buildings, this parameter increases significantly and is highly dependent on environmental conditions.

## 3. Results

**Figure 7:**
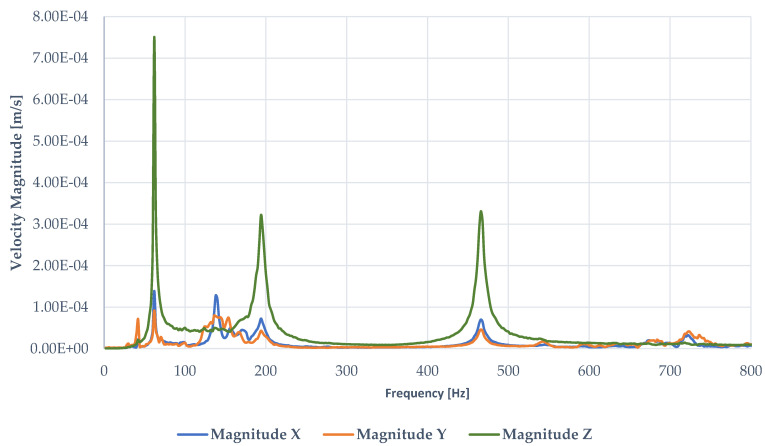
FFT results from measurements of velocity magnitude—measurement of the full surface scan by 3D LDV in the range of 0–800 Hz.

The surface scan was performed for 120 points located as shown in Figure 5 (right). Each point was remeasured three times to additionally increase the quality of data and the postprocess animation. Figure 7 presents the results of the overall frequency response functions (velocity magnitude). The data were obtained after averaging over all measurement points of the full surface after the software excluded the points of insufficient quality.

**Figure 8:**
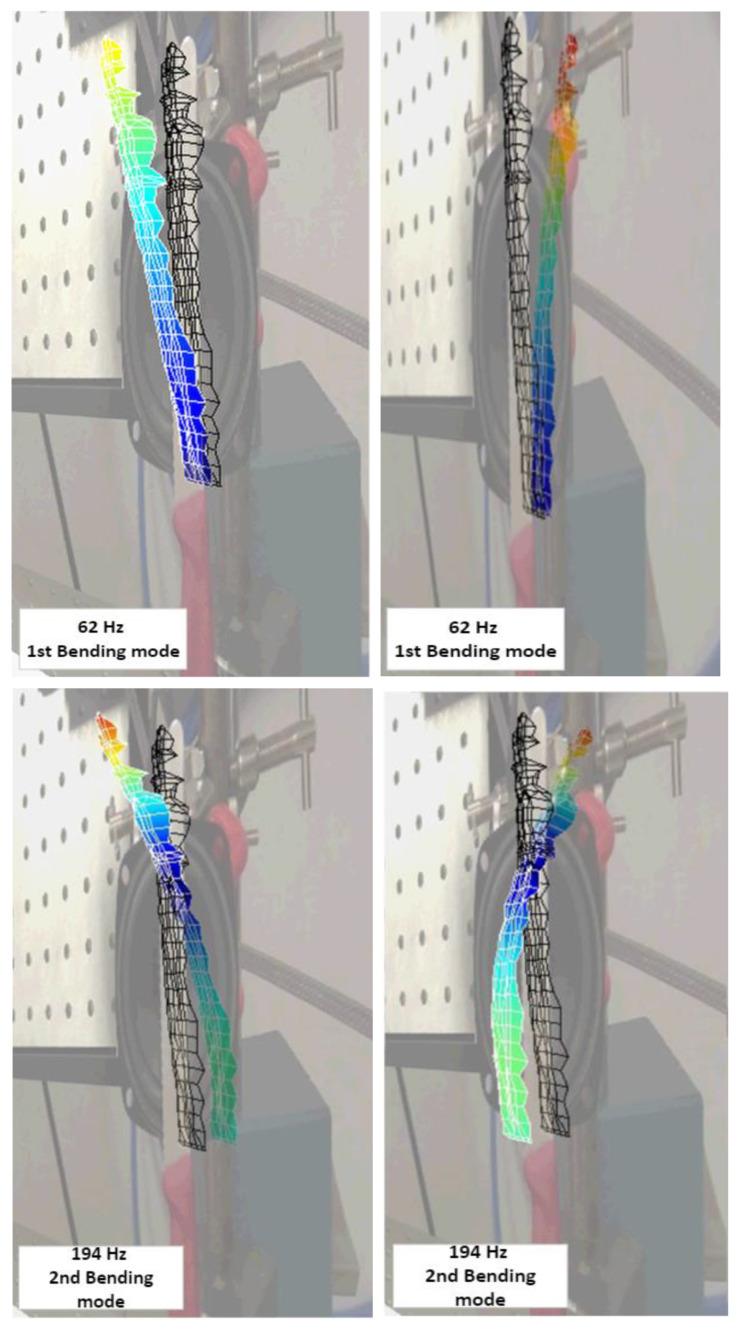

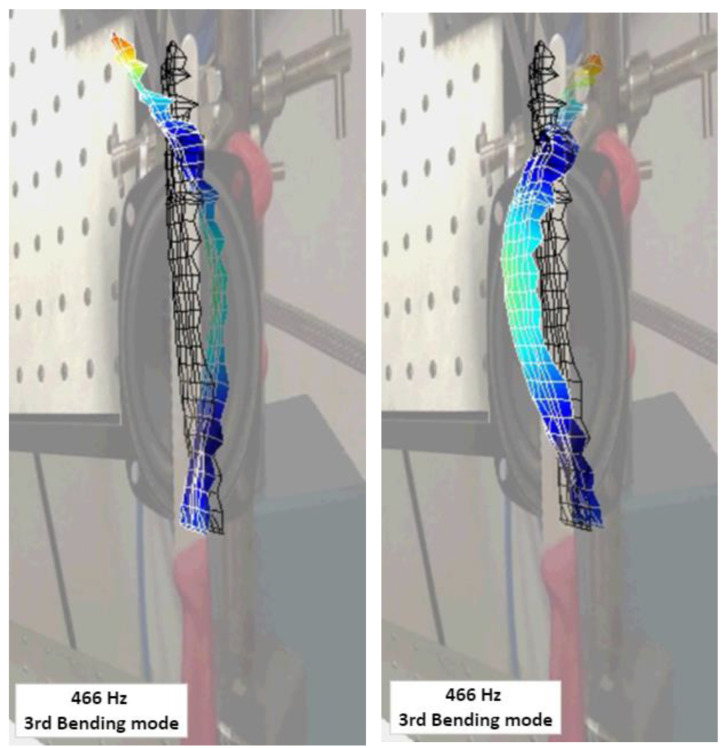
Mode shape for the three first bending modes at 62, 194, and 466 Hz (data visualization from 3D Laser Vibrometer).

Figure 8 shows static images of the first three bending modes taken by the scanner system (also the green line picks in Figure 7). The measurement was made from several surface points, and the final result is the visualization of the movement of all points. In this way, the observer can exclude points that do not behave similarly to others (in terms of amplitude or direction of movement). Thanks to the capabilities of the software, additional mathematical operations are also possible, which allow better visualization of the final spatial form. The animation can also be superimposed with the original image from the main camera of the vibrating head. This approach improves the user’s understanding of the data analysis and allows a correct assessment of the mode shape type.

The data can be presented in multiple ways. The main one is creating an FRF plot for each point or averaged plot for all surface points. Additionally, the data can be extracted and used in many other formats and in both commercial (Labview, MeScope, Matlab, etc.) and free systems. The additional programs and plug-ins, e.g., Python codes, can also be incorporated to send the data to production or companies’ distributed cloud software and solutions (SCADA, MES, ERP, etc.). Then, the data can be directly applied for QA and QC analysis, with the data and results stored in the chosen software. 

In addition, Table 1 shows the natural frequencies and modal shapes of the investigated steel blade in the range of 0–1.1 kHz. Thanks to the three measuring heads of the Laser Doppler Vibrometry System (PSV-500), it is also possible to detect the directional dependence of each vibration mode. Thus, not only the frequency but also the type of modal shape can be detected and later animated. The frames presenting maximal curvatures are presented in Figure 8.

## 4. Discussion and Significance of Results for Industrial QA and QC

### 4.1. Results’ Significance

It is worth mentioning that the laser sensor in each of the heads allows an angular resolution of <0.001°, an angular stability of <0.001°/h, and up to 30 scan points/s. This last parameter is especially useful while using the system in continuous measurements on a production line without stopping the process for quality checks. Single-point measurement is possible in just 30 ms. Additionally, if there is no change in position between each product on the production line, there is no need to perform preparation tasks such as 2D alignment, 3D alignment, scan point assignment, and laser beam focusing. This makes the system perfect for QA and QC on the production line.

The author proposes a multilevel system for QA and QC based on the 3D Laser Vibrometer:

- First layer: The sensing platform where the system can operate independently performing 2D (single-point) or 3D (surface) measurements and providing warning information if the data does not match the template product. This layer also allows communication with higher-level software.
- Second layer: The middleware where the database system is implemented and information can be stored in the cloud for future analysis. For production management, it can be system control and data acquisition software (SCADA) in the case of production control, and product data management (PDM) or product lifecycle management (PLM) in the case of product information management (design, prototyping, volume production, revisions, lifecycle tracking, etc.). This layer also allows communication with decentralized platforms and enterprise services.
- Third layer: The distributed system level where whole company data can be analyzed, stored and compared. With the use of modern technologies (big data, blockchain), the data can be used in a wider range of areas, e.g., to optimize production processes and limit the number of production faults.

**Figure 9:**
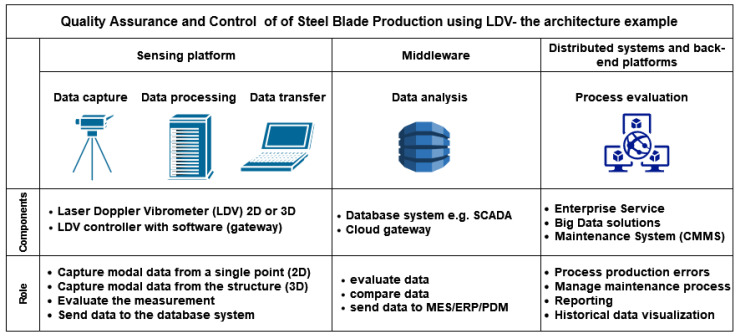
The proposed approach to QA and QC with 3D LDV.

Figure 9 presents the overall architecture of data acquisition and its usage in the case of the production process.

**Figure 10:**
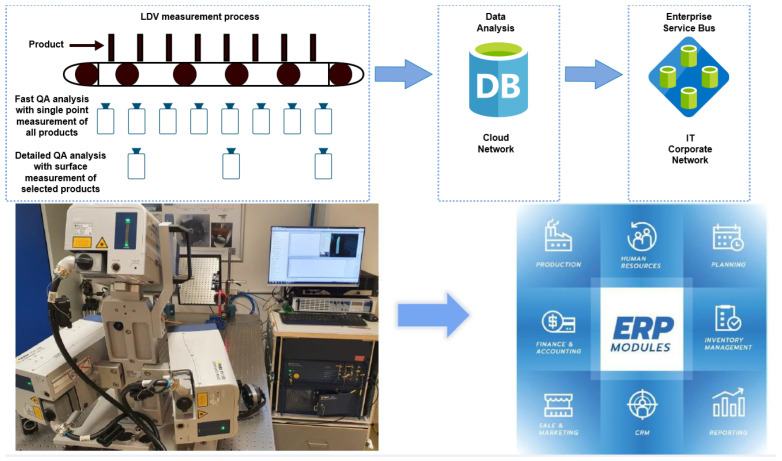
Incorporation of 3D LDV in QA and QC for Industry 4.0.

In most QA and QC implementations, only randomly chosen elements, sets, or production series are chosen for control purposes. However, if the measurement can be limited to only 30 ms, this can be performed on the go without losing time for each of the products, thereby limiting the number of faulty products sent to companies’ clients. Figure 10 presents the proposed QA process where each element is tested with a single-point measurement and every n-product is tested with a surface scan (few minutes’ measurement). Data for every single point and surface measurement can then be stored in the cloud for comparison purposes and later can be analyzed in the enterprise services.

To allow a data comparison (e.g., using cloud solutions), some kind of evaluation algorithm is necessary. The chosen algorithm will strongly depend on the field of usage and the sensor used. In the following chapter, a possible solution for on-the-go comparison with the template will be presented.

### 4.2. Proposed QA Algorithm and Evaluation Process

Due to its capability for quick measurements, the QA process will require some kind of performance evaluation criterion. The proposed method uses an algorithm based on the comparison of the benchmark product ($LDV_{ref}$) with the specific product ($LDV_{product}$). This case does not require the person responsible for the QA process to do anything more than define the level of acceptable positive evaluation for the product.

By using the software provided with the LDVs, a cloud-based application, or the SCADA system for the production line, the QA engineer can calculate the maximal difference (D1) and RMS difference (D2) between the two readings. Using each scan point’s value, the maximum difference is calculated. In the case of ‘n’ scan points, the difference is calculated using Equation (7).

(7) $$D_{1}\left(i\right)=\left(abs\;\left(LDV_{ref}\left(i\right)-LDV_{product}\left(i\right)\right)\right)$$

Thus, the maximum percentage difference is calculated from (8).

(8) $$D_{1}\left(\%\right)=\left(\frac{\max\left(D_{1}\right)}{\max\left(abs\left(LDV_{ref}\right)\right)}\right)*100$$

Alternatively, it is convenient to calculate RMS differences for ‘n’ sampling points from (9).

(9) $$RMS_{Diff}=\sqrt{\frac{\sum_{i=1}^{n}{\left(LDV_{ref}\left(i\right)-LDV_{product}\left(i\right)\right)}^{2}}{n}}$$

As the last step, the RMS difference normalized by the range can be calculated by (10).

(10) $$D_{2}\left(\%\right)=\left(\frac{RMS_{Diff}}{\max\left(LDV_{ref}\right)-\min\left(LDV_{ref}\right)}\right)*100$$

A similar method, but for the overall performance assessment of measurement quality for civil engineering structures and the validation of displacement sensors, was used by Gomez et al. [42] and Garg [43] with good results. Thus, the algorithm presented is a known algorithm for the assessment of the displacement of civil engineering structures. However, the typical usage is with the implementation of typical displacement sensors or accelerometers in the case of dynamic measurements. The proposed approach incorporates a novel optical measurement system, the 3D Laser Doppler Vibrometer. Additionally, the implementation in the previous usage of this algorithm was for dynamic measurements of the structures under typical excitations (traffic). The proposed approach is the implementation of such a system and algorithm for ultra-fast measurements (just a few milliseconds) that allows it to be used also for QA and QC. This is a novel approach.

## 5. Conclusions

In summary, dynamic measurements play a critical role in the design and analysis of equipment and quality control. Moreover, the use of novel, optical techniques like 3D laser vibration measurement systems in quality control processes, especially quality assurance and quality control as part of an Industry 4.0 strategy, is very advantageous. The QA&C engineer can measure and compare the frequency response of mass-production products. The potential use of non-contact excitation allows such systems to be implemented in production lines to perform product quality assessments without additional stops and human involvement. This single-point measurement takes only a few milliseconds and can be synchronized with the flow of the production process. Moreover, due to the globalization effect, one company can have factories in different parts of the world, but the quality of the same products must be the same, and in many cases the clients require proof of quality. With cloud-based solutions and the possibility of easy data storage and comparison, this can be assured smoothly with the use of the presented system. This paper also presents a simple QA comparison algorithm that can be used in such a process. Future research will focus on connecting the 3D LDV production data with AI solutions for predictive maintenance.

## Figures and Tables

**Table 1:** Overall results of the modal analysis in the 0–1.1 kHz range.

Mode Number	Modal Shape	Frequency (Hz)
1	1st Bending	62
2	1st Torsional	138
3	2nd Bending	194
4	3rd Bending	466
5	1st Lateral	722
6	4th Bending	904
7	2nd Torsional	1004

## Data Availability

Not applicable.
